# Ischemic proctitis 6 months after laparoscopic sigmoidectomy: a case report

**DOI:** 10.1186/s40792-021-01133-7

**Published:** 2021-02-22

**Authors:** Takuto Yoshida, Nobuki Ichikawa, Shigenori Homma, Tadashi Yoshida, Shin Emoto, Yoichi Miyaoka, Hiroki Matsui, Akinobu Taketomi

**Affiliations:** Department of Gastroenterological Surgery I, Graduate School of Medicine, Hokkaido University, W-7, Kita-ku, Sapporo, N-15060-8638 Japan

**Keywords:** Ischemic proctitis, Ischemic colitis, Sigmoid colon cancer, Bloody stool, Laparoscopic sigmoidectomy, Non-surgical management, Hyperbaric oxygen therapy

## Abstract

**Background:**

Ischemic colitis is a common disease; however, its pathophysiology remains unclear, especially in ischemic proctitis after sigmoidectomy. We present a rare case of ischemic proctitis 6 months after laparoscopic sigmoidectomy.

**Case presentation:**

The patient was a 60-year-old man with hypertension, type 2 diabetes, and hyperlipidemia. He was a smoker. He underwent laparoscopic sigmoidectomy for pathological stage I sigmoid colon cancer and was followed up without any adjuvant therapy. Six months after his surgery, he complained of lower abdominal discomfort, bloody stools, and tenesmus. Colonoscopy showed extensive rectal ulcers between the anastomotic site and the anal canal, which was particularly severe on the anal side several centimeters beyond the anastomosis. We provided non-surgical management, including hyperbaric oxygen therapy. The rectal ulcers had healed 48 days after the therapeutic intervention. He has not experienced any recurrence for 3.5 years.

**Conclusions:**

While performing sigmoidectomy, it is important to consider the blood backflow from the anal side of the bowel carefully, especially for patients with risk factors of ischemic proctitis.

## Background

Ischemic colitis is a common disease, and ischemic proctitis after sigmoidectomy is rare. The pathophysiology and definitive management remain unclear. We present a case of ischemic proctitis that occurred 6 months after laparoscopic sigmoidectomy and treated by non-surgical management.

## Case presentation

**Fig. 1:**
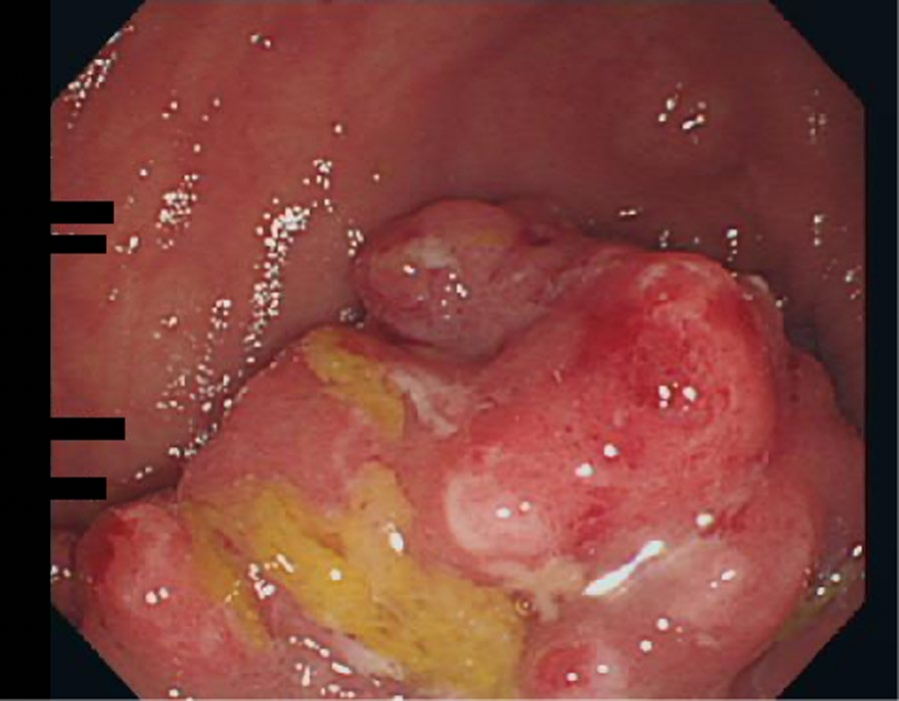
Colonoscopy findings. Type 1 tumor in the sigmoid colon. A biopsy of the lesion reveals well-differentiated adenocarcinoma

**Fig. 2:**
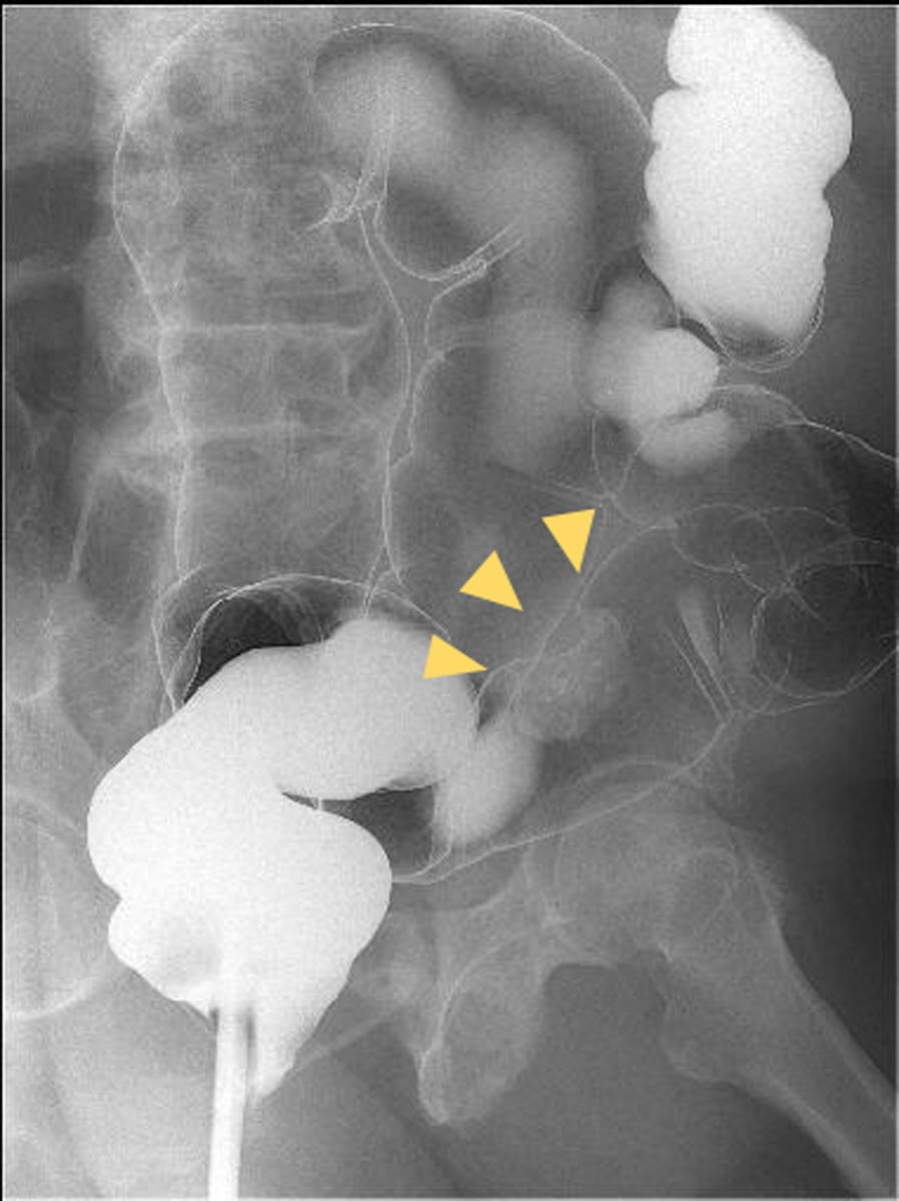
Radiographic contrast enema findings. A 3-cm stricture can be seen in the sigmoid colon, and the apple core sign is positive (yellow arrow)

**Fig. 3:**
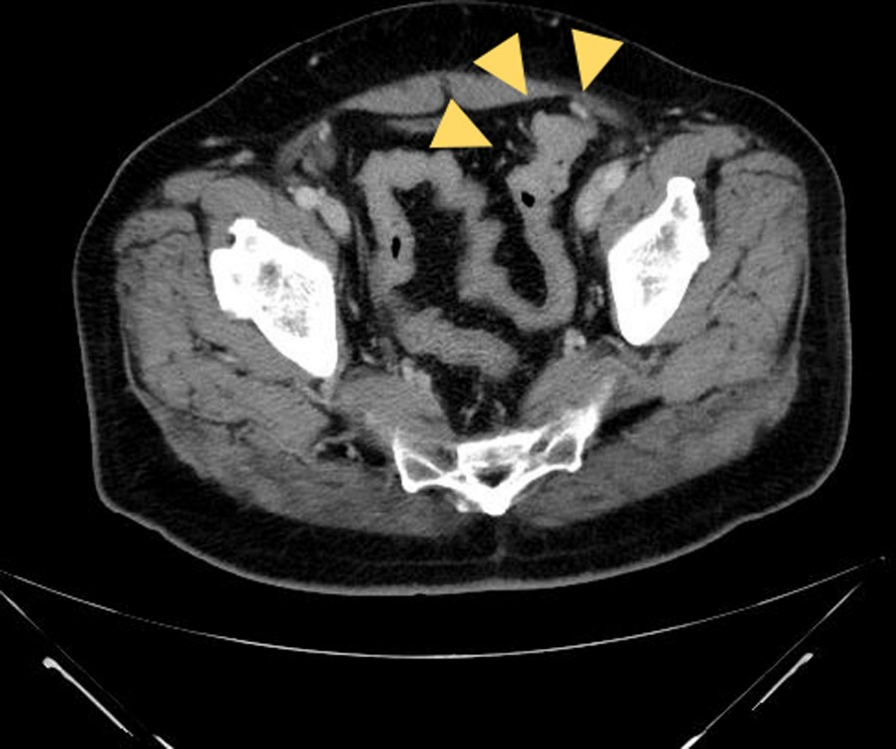
Abdominal computed tomography findings. Abdominal computed tomography image shows a contrast-enhanced lesion in the sigmoid colon (yellow arrow). There are no metastases or ascites

**Fig. 4:**
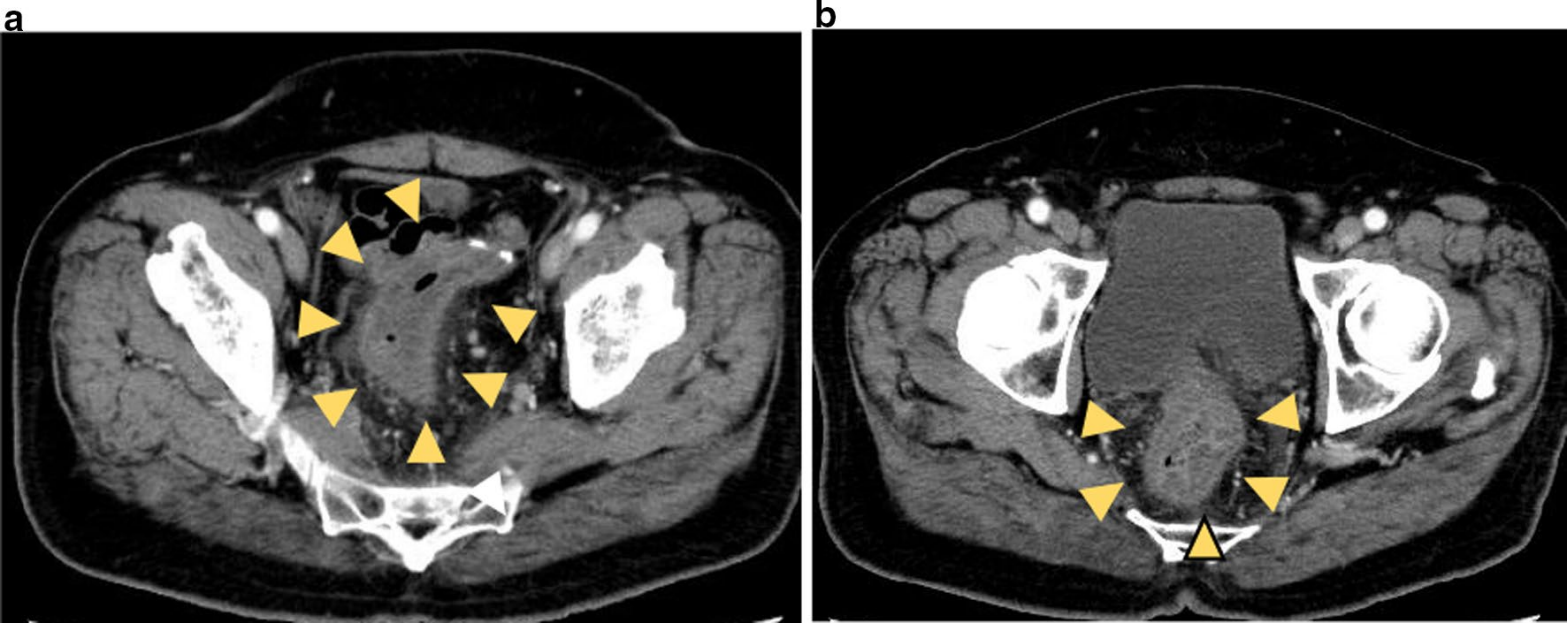
Abdominal computed tomography findings. The images show edematous changes in the anal side of the anastomosis (yellow arrow). **a** The severest part of the ischemic lesion is not located adjacent to, but **b** is located at a short distance from the anastomosis towards the anal canal

**Fig. 5:**
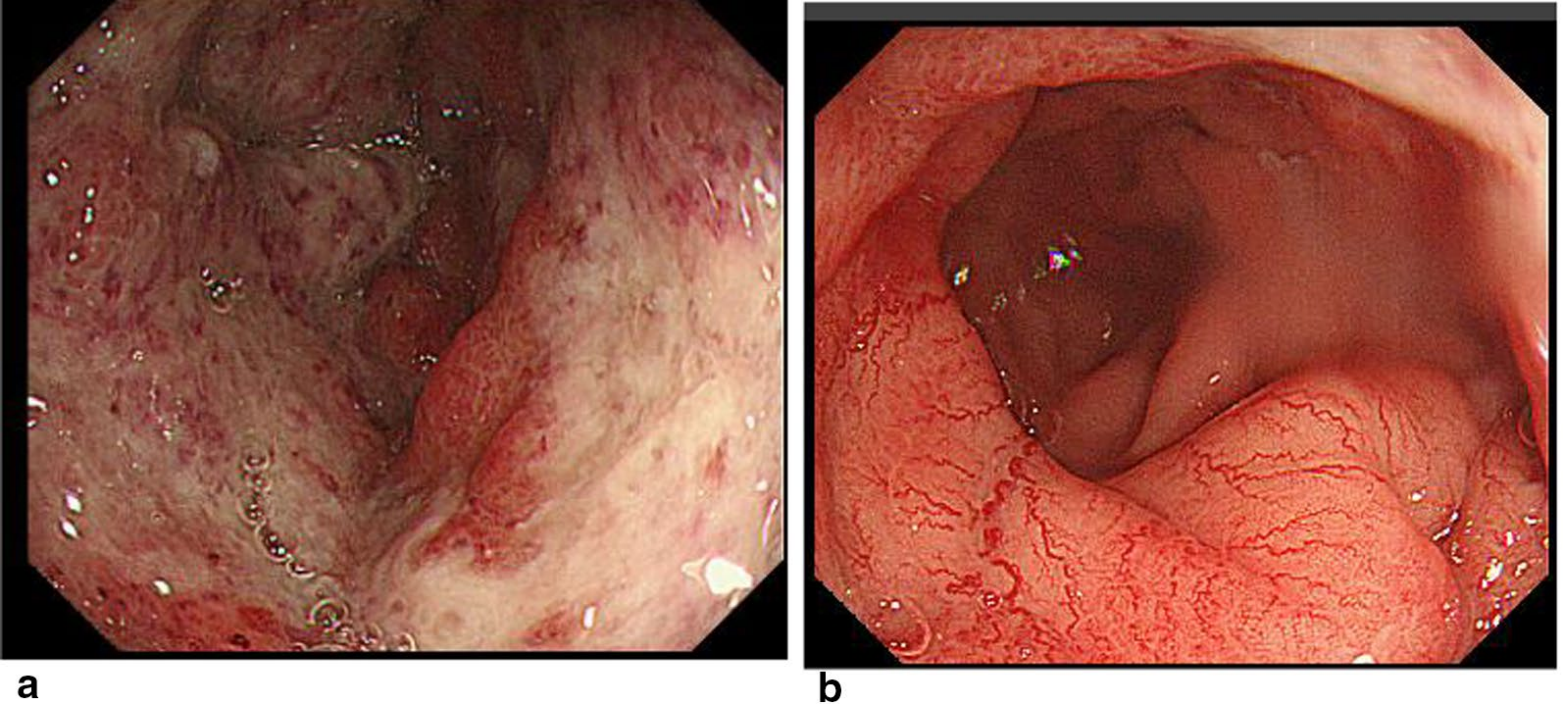
Colonoscopy findings. **a** A circumferential ulcer is seen not adjacent to but near the anal side of the anastomosis. **b** The mucosa around the anal canal appears intact

**Fig. 6:**
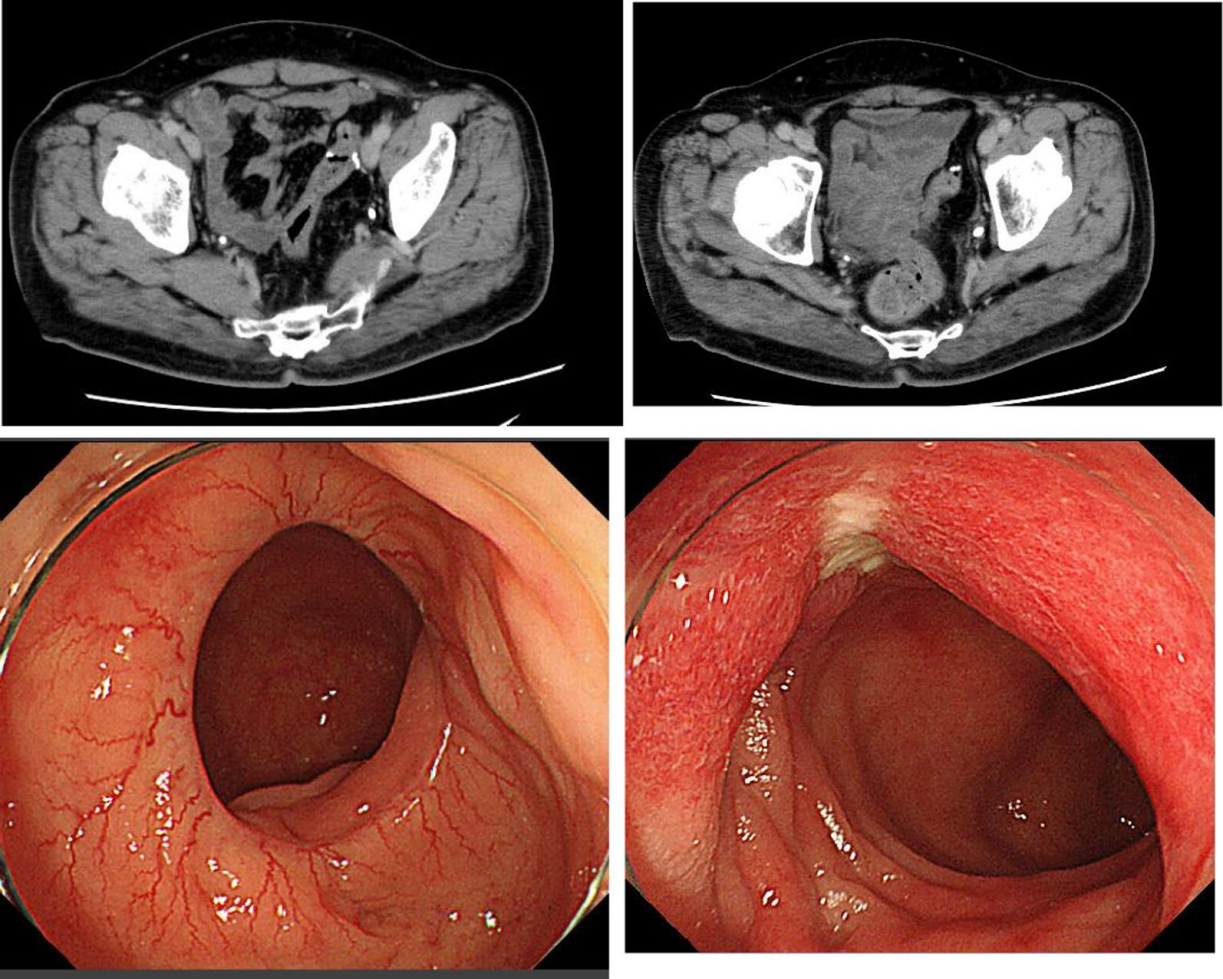
Computed tomography and colonoscopy findings after non-operative management. Computed tomography was performed on the 46th day and colonoscopy on the 35th day. Edematous changes and circumferential ulcers show improvement

A 60-year-old man underwent an occult bleeding test during his annual medical checkup, which showed positive results. Colonoscopy showed a type 1 tumor in the sigmoid colon (Fig. 1). The biopsy of the lesion showed well-differentiated adenocarcinoma. The patient had hypertension, type 2 diabetes, and hyperlipidemia. He had a history of smoking half a pack of cigarettes per day for 27 years, which he quit 15 years back. His family history was unremarkable. On physical examination, his abdomen was soft and non-distended. Laboratory data showed carcinoembryonic antigen (CEA) 6.1 ng/mL and carbohydrate antigen (CA)19–9 52 U/mL. Radiographic contrast enema showed an approximately 3-cm stricture in the sigmoid colon, and the apple core sign was positive. It was located between the upper part of the sigmoid colon and descending colon. The sigmoid colon was redundant (Fig. 2). Abdominal computed tomography (CT) showed a contrast-enhanced lesion in the sigmoid colon without metastases (Fig. 3). He underwent laparoscopic sigmoidectomy with D3 lymph node dissection. The inferior mesenteric artery was ligated high. The sigmoid colon was resected at the level of the promontory, about 30 cm from the anal verge. We performed a double-stapling technique for the anastomosis. The length of resected specimen was 250 mm, and the tumor was located 200 mm from the distal margin. The surgical time was 135 min, and blood loss was 10 mL. His postoperative course was normal, and he was discharged on postoperative day 11. The pathology was S, type 2, 35 × 30 mm, adenocarcinoma (tub2 > tub1), T2 (MP), N0, M0 pStage I. He was followed up without any adjuvant therapy. Six months after his surgery, he presented with mild lower abdominal discomfort, tenesmus, and bloody stools. Abdominal CT revealed extensive edematous changes in the rectum between the anastomotic site and the anal canal (Fig. 4). His symptoms had been deteriorating for 1 week with anal pain. Therefore, he was admitted to our department. His white blood cell count was 10,100/μL, and C-reactive protein was 2.04 mg/dL. Colonoscopy showed extensive circumferential rectal ulcers between the anastomotic site and the anal canal, which was especially severe and several centimeters beyond the anastomosis on the anal side. The mucosa around the anal canal was intact (Fig. 5). The stool culture was negative. The biopsy of the lesion showed erosive and regenerative colonic mucosa, and there was no malignancy or cytomegalovirus infection. He was diagnosed with ischemic proctitis and administered antibiotics. Since the patient did not have any peritoneal signs, we selected a non-surgical treatment. Oral intake was stopped. We started with 10 sessions of hyperbaric oxygen therapy (HBO) for the patient on day 3 of admission. Prostaglandin E1 (PGE1) and Kampo medicines (®Saireito) were started on day 5, and nitric oxide (NO) was started on day 12. Oral intake was resumed on day 4. His rectal inflammation improved, and the last CT scan and colonoscopy showed normal findings 46 days after admission (Fig. 6). He was discharged on day 48. He continues to take PGE1 and has used NO therapy for 1 year. He has remained symptom-free for 3.5 years.

## Discussion

Ischemic colitis is defined as a localized and reversible ischemic colonic lesion [1, 2] that usually occurs at the ileocecal junction and around the Griffith’s point and Sudeck’s point [3]. Ischemic proctitis occurs in only 2–5% of cases of ischemic coloproctitis because of the abundant collateral blood supply of the rectum [4, 5]. Moreover, ischemic proctitis after sigmoidectomy is rare [6]. There have been no reports on the prevalence of ischemic proctitis after sigmoidectomy. In general, the rectum is uncommonly affected by ischemia due to its relatively rich dual blood supply from both systemic and splanchnic circulation [7]. Bharucha et al. reported 328 ischemic colitis patients; of these, only 10 patients had isolated ischemic proctosigmoiditis [8]. In the present case, insufficient blood flow due to dissection of the inferior mesenteric artery may have contributed to the development of ischemic proctitis. Nelson et al. reported that an incidence of ischemic colitis after abdominal aortic surgery with inferior mesenteric artery resection is at least 0.5%, suggesting that ischemic proctitis is very rare [9]. Here, we presented a rare case of ischemic proctitis that occurred 6 months after laparoscopic sigmoidectomy. The pathophysiology of ischemic proctitis after sigmoidectomy remains unclear. However, it was assumed to be related to several factors, including arteriosclerosis [9–12], venous congestion [13, 14], and anatomically complicated areas such as the Sudeck’s point [3, 15–18]. Sudeck’s point is a watershed area and a weak blood supply point at the rectosigmoid junction [15, 17]. An incomplete vascular anastomosis at the Sudeck’s point would result in ischemic changes in the remaining rectosigmoid colon. This could cause anastomotic leakage after sigmoidectomy [18]. This patient's anastomotic site was at the sigmoid colon approximately 10 cm higher than the promontorium level with high ligation of the inferior mesenteric artery, which might be one of the causes of impaired vascular perfusion. However, the severest part of the ischemic lesion was not located at the anastomosis and the ulcers were spread extensively towards the anal canal. Additionally, the patient did not experience anastomotic leakage, which suggests that the Sudeck’s point was not the sole cause of this patient’s proctitis. We assume that the blood insufficiency was partial. Some inflammation in the underlying arteriosclerotic disease might have combined with the anatomical problem. Therefore, based on the present case, we believe that the blood backflow from the anal side should be considered more carefully, especially in patients with risk factors for ischemic proctitis such as arteriosclerotic disease. Evaluation of blood flow in the anastomotic bowel using indocyanine green is considered one of the methods for preventing bowel ischemia and anastomotic leakage [19]. Besides, since the ischemic proctitis occurred 6 months after sigmoidectomy in this case, intraoperative blood flow evaluation alone might not be sufficient as a preventive method. Considering the retrograde blood flow from the rectum, the most important thing is that the colon should not be resected distant from the anal verge in cases with redundant sigmoid colon.

The first-line treatment for ischemic colitis is non-surgical management, including withholding oral intake and administration of antibiotics. In severe cases, surgery including colostomy or abdominoperineal resection is indicated [20]. In general, O’Neill et al. reported that aggravating risk factors of ischemic colitis include lack of rectal bleeding, peritonism, renal dysfunction, and right-sided ischemic colitis [21]. The patient did not have any peritoneal signs or aggravating risk factors. Furthermore, the patient’s abdominal symptoms improved relatively fast with bowel rest, and surgical treatment was unnecessary. If the patient had abdominal discomfort with peritoneal signs or conservative treatment was unsuccessful, we would have performed the surgery. For non-surgical management, there have been reports of the use of HBO [22], vasodilators such as PGE1 [23], and NO [24]. The main aim of non-surgical management of ischemic proctitis is restoring tissue perfusion. Tissue perfusion is classified into three groups, namely, adequate perfusion, no perfusion, and marginal perfusion, called penumbra [25]. Ischemic-reperfusion injury induces leukocyte adherence to endothelial cells where they are activated. This activation results in the release of reactive oxygen species, which convert xanthine dehydrogenase into xanthine oxidase necessary for lipid peroxidation. This reaction induces tissue and organ injuries. In addition to tissue oxygenation and reducing edema via vasoconstriction [26], HBO inhibits β2 integrin-mediated leukocyte adherence to endothelial cells in the animal model and decreases ischemic-reperfusion injury; thus, HBO exerts its greatest influence in the penumbra [27]. In the present case, there was no mechanical vessel obstruction, and the anastomotic site was also considered to be the penumbra; therefore, HBO, which decreases the effect of ischemic-reperfusion injury, was effective in this case. Patients without aggravating risk factors are good candidates for non-surgical management, including HBO.

## Conclusions

Ischemic proctitis after sigmoidectomy might be related to underlying arteriosclerosis and anatomical blood flow insufficiency. It is important to carefully consider the blood backflow from the anal side, especially in patients with risk factors for ischemic proctitis.

## Data Availability

The dataset supporting the conclusions of this article is included in the article.

## Abbreviations

CA19-9: Carbohydrate antigen 19-9
CEA: Carcinoembryonic antigen
CT: Computed tomography
HBO: Hyperbaric oxygen therapy
NO: Nitric oxide
PGE1: Prostaglandin E1
